# Bis­(3-*tert*-butyl­pyridine-κ*N*)bis­(4-*tert*-butyl­pyridine-κ*N*)bis(thio­cyanato-κ*N*)cadmium

**DOI:** 10.1107/S1600536812040081

**Published:** 2012-10-20

**Authors:** Thorben Reinert, Inke Jess, Christian Näther

**Affiliations:** a Institut für Anorganische Chemie, Christian-Albrechts-Universität Kiel, Max-Eyth Strasse 2, D-24098 Kiel, Germany

## Abstract

The asymmetric unit of the title compound, [Cd(NCS)_2_(C_9_H_13_N)_4_], consists of one Cd^II^ cation located on a centre of inversion, one thio­cyanate anion, one 3-*tert*-butyl­pyridine ligand and one 4-*tert*-butyl­pyridine ligand in general positions. The *tert*-butyl group of the 4-*tert*-butyl­pyridine ligand is disordered over two sets of sites in a 0.25:0.75 ratio and was refined using a split model. The Cd^II^ cation is coordinated by six N atoms of four *tert*-butyl­pyridine ligands and two *N*-bonded thio­cyanate anions within a slightly distorted octa­hedral coordination environment.

## Related literature

For the synthesis and properties of coordination polymers based on transition metal thio­cyanates and *N*-donor ligands, see: Boeckmann & Näther (2010 ▶, 2011 ▶). For related structures, see: Nassimbeni *et al.* (1990 ▶) (4-*tert*-butyl­pyridine only).
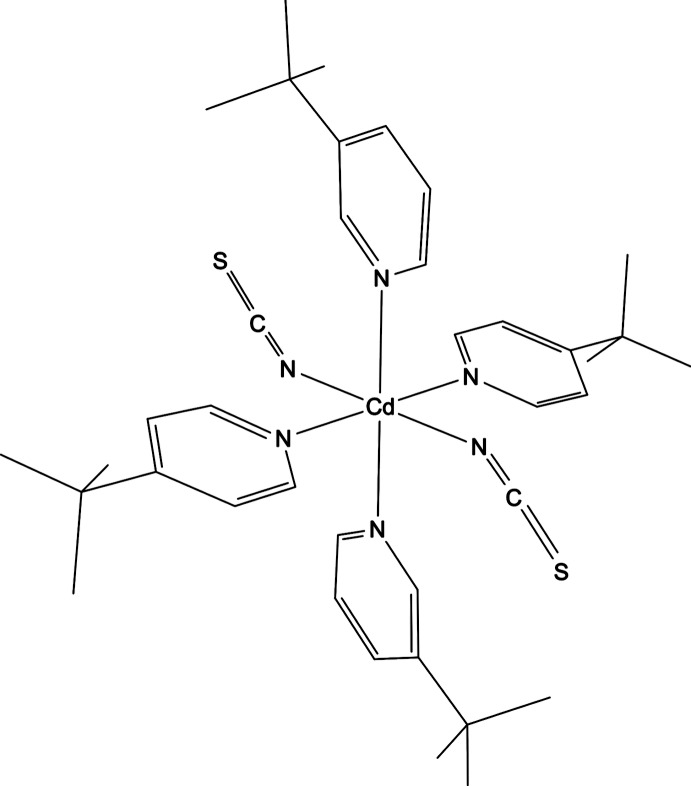


## Experimental

### 

#### Crystal data

[Cd(NCS)_2_(C_9_H_13_N)_4_]
*M* *_r_* = 769.38
Triclinic, 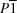

*a* = 9.5136 (7) Å
*b* = 10.7582 (7) Å
*c* = 11.6674 (10) Å
α = 67.142 (8)°
β = 68.242 (9)°
γ = 76.472 (8)°
*V* = 1016.32 (13) Å^3^
*Z* = 1
Mo *K*α radiation
μ = 0.67 mm^−1^
*T* = 200 K
0.16 × 0.11 × 0.07 mm

#### Data collection

Stoe IPDS-1 diffractometer
11989 measured reflections
4709 independent reflections
4109 reflections with *I* > 2σ(*I*)
*R*_int_ = 0.077

#### Refinement

*R*[*F* ^2^ > 2σ(*F* ^2^)] = 0.051
*wR*(*F* ^2^) = 0.127
*S* = 1.02
4709 reflections
226 parameters
3 restraints
H-atom parameters constrained
Δρ_max_ = 1.69 e Å^−3^
Δρ_min_ = −1.58 e Å^−3^

### 

Data collection: *X-AREA* (Stoe & Cie, 2008 ▶); cell refinement: *X-AREA*; data reduction: *X-AREA*; program(s) used to solve structure: *SHELXS97* (Sheldrick, 2008 ▶); program(s) used to refine structure: *SHELXL97* (Sheldrick, 2008 ▶); molecular graphics: *XP* (Sheldrick, 2008 ▶) and *DIAMOND* (Brandenburg, 2011 ▶); software used to prepare material for publication: *publCIF* (Westrip, 2010 ▶).

## Supplementary Material

Crystal structure: contains datablock(s) I, global. DOI: 10.1107/S1600536812040081/bt6836sup1.cif

Structure factors: contains datablock(s) I. DOI: 10.1107/S1600536812040081/bt6836Isup2.hkl

Additional supplementary materials:  crystallographic information; 3D view; checkCIF report

## Figures and Tables

**Table 1:** Selected bond lengths (Å)

Cd1—N1^i^	2.301 (3)
Cd1—N21^i^	2.375 (3)
Cd1—N11^i^	2.403 (3)

Symmetry code: (i) 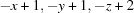.

**Fig. 1.**
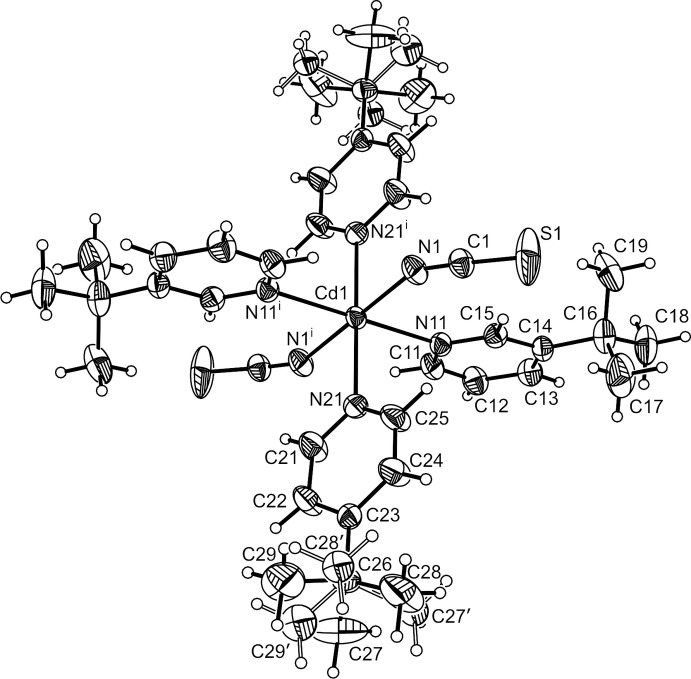
Crystal structure of the title compund with labelling and displacement ellipsoids drawn at the 50% probability level. Symmetry codes: i = -x + 1, -y + 1, -z + 2. Disordering is shown as full and open bonds.

## supplementary crystallographic information

### Comment

The structure determination of the title compound was performed as a part of a project on the synthesis and properties of new coordination polymers based on transition metal thiocyanates and and N-donor ligands (Boeckmann & Näther, 2010, 2011). Within this project we have reacted cadmium(II)thiocyanate with 4-*tert*-butylpyridine in water, which results in the formation of crystals of the title compound by accident. Apparently, the 4-*tert*-butylpyridine was contaminated with 3-*tert*-butylpyridine to a degree that allowed the formation of a few single crystals.

In the crystal structure the Cd atoms are coordinated by six N atoms of four *tert*-butylpyridine ligands and two N-bonded thiocyanato anions in mutual *trans* orientation in a slightly distorted octahedral geometry (Fig. 1 and Table 1). The Cd···N distances range from 2.3005 (36) Å to 2.4025 (28) Å. It is also worth mentioning that so far no other compound containing 3-*tert*-butylpyridine has been reported in the CSD.

### Experimental

The title compound was obtained accidently during the reaction of 34.3 mg Cd(NCS)_2_ (0.15 mmol) with 88.8 µl 4-*tert*-butylpyridine (0.60 mmol) in 1.50 ml water at RT in a closed 3 ml snap cap vial. After two weeks colourless needles of the title compound were obtained.

### Refinement

The H atoms were positioned with idealized geometry and were refined using a riding model with *U*_iso_(H) = 1.2*U*_eq_(C) (1.5 for methyl H atoms) of the parent atom using a riding model with C—H = 0.95 and 0.98 Å. The *tert*-butyl group of the 4-*tert*-butylpyridine ligand is disordered and was refined using a split model with fixed site occupation factors of 0.75 and 0.25. The distances between the methyl groups in the two disordered moieties were restrained to be equal.

### Crystal data

**Table:**

[Cd(NCS)_2_(C_9_H_13_N)_4_]	*Z* = 1
*M**_r_* = 769.38	*F*(000) = 402
Triclinic, *P*1	*D*_x_ = 1.257 Mg m^−^^3^
Hall symbol: -P 1	Mo *K*α radiation, λ = 0.71073 Å
*a* = 9.5136 (7) Å	Cell parameters from 8000 reflections
*b* = 10.7582 (7) Å	θ = 1.9–28.2°
*c* = 11.6674 (10) Å	µ = 0.67 mm^−^^1^
α = 67.142 (8)°	*T* = 200 K
β = 68.242 (9)°	Needle, colourless
γ = 76.472 (8)°	0.16 × 0.11 × 0.07 mm
*V* = 1016.32 (13) Å^3^	

### Data collection

**Table:**

Stoe IPDS-1 diffractometer	4109 reflections with *I* > 2σ(*I*)
Radiation source: fine-focus sealed tube	*R*_int_ = 0.077
Graphite monochromator	θ_max_ = 28.0°, θ_min_ = 2.3°
φ scans	*h* = −12→12
11989 measured reflections	*k* = −13→13
4709 independent reflections	*l* = −15→15

### Refinement

**Table:**

Refinement on *F*^2^	Primary atom site location: structure-invariant direct methods
Least-squares matrix: full	Secondary atom site location: difference Fourier map
*R*[*F*^2^ > 2σ(*F*^2^)] = 0.051	Hydrogen site location: inferred from neighbouring sites
*wR*(*F*^2^) = 0.127	H-atom parameters constrained
*S* = 1.02	*w* = 1/[σ^2^(*F*_o_^2^) + (0.0811*P*)^2^] where *P* = (*F*_o_^2^ + 2*F*_c_^2^)/3
4709 reflections	(Δ/σ)_max_ < 0.001
226 parameters	Δρ_max_ = 1.69 e Å^−^^3^
3 restraints	Δρ_min_ = −1.58 e Å^−^^3^

### Special details

**Table:**

Geometry. All e.s.d.'s (except the e.s.d. in the dihedral angle between two l.s. planes) are estimated using the full covariance matrix. The cell e.s.d.'s are taken into account individually in the estimation of e.s.d.'s in distances, angles and torsion angles; correlations between e.s.d.'s in cell parameters are only used when they are defined by crystal symmetry. An approximate (isotropic) treatment of cell e.s.d.'s is used for estimating e.s.d.'s involving l.s. planes.
Refinement. Refinement of *F*^2^ against ALL reflections. The weighted *R*-factor *wR* and goodness of fit *S* are based on *F*^2^, conventional *R*-factors *R* are based on *F*, with *F* set to zero for negative *F*^2^. The threshold expression of *F*^2^ > σ(*F*^2^) is used only for calculating *R*-factors(gt) *etc*. and is not relevant to the choice of reflections for refinement. *R*-factors based on *F*^2^ are statistically about twice as large as those based on *F*, and *R*-factors based on ALL data will be even larger.

### Fractional atomic coordinates and isotropic or equivalent isotropic displacement parameters (Å^2^)

**Table:**

	*x*	*y*	*z*	*U*_iso_*/*U*_eq_	Occ. (<1)
Cd1	0.5000	0.5000	1.0000	0.02671 (12)	
N1	0.6536 (4)	0.6715 (3)	0.9156 (3)	0.0392 (7)	
C1	0.6855 (4)	0.7786 (4)	0.8456 (4)	0.0362 (7)	
S1	0.73133 (15)	0.92652 (13)	0.74250 (19)	0.0869 (6)	
N11	0.2957 (3)	0.6619 (3)	0.9404 (3)	0.0302 (6)	
C11	0.1577 (4)	0.6192 (4)	0.9843 (4)	0.0371 (7)	
H11	0.1461	0.5265	1.0353	0.044*	
C12	0.0319 (4)	0.7042 (4)	0.9587 (4)	0.0419 (8)	
H12	−0.0639	0.6702	0.9903	0.050*	
C13	0.0472 (4)	0.8402 (4)	0.8861 (4)	0.0384 (8)	
H13	−0.0383	0.8999	0.8669	0.046*	
C14	0.1875 (4)	0.8890 (3)	0.8415 (3)	0.0316 (7)	
C15	0.3087 (4)	0.7938 (3)	0.8709 (3)	0.0322 (7)	
H15	0.4063	0.8246	0.8396	0.039*	
C16	0.2138 (4)	1.0380 (4)	0.7633 (4)	0.0449 (9)	
C17	0.3189 (7)	1.0495 (6)	0.6244 (5)	0.0775 (18)	
H17A	0.3363	1.1446	0.5740	0.116*	
H17B	0.2713	1.0161	0.5819	0.116*	
H17C	0.4163	0.9951	0.6284	0.116*	
C18	0.0626 (6)	1.1273 (5)	0.7600 (6)	0.0638 (13)	
H18A	0.0827	1.2220	0.7100	0.096*	
H18B	−0.0022	1.1199	0.8496	0.096*	
H18C	0.0110	1.0971	0.7182	0.096*	
C19	0.2904 (5)	1.0892 (5)	0.8294 (6)	0.0611 (13)	
H19A	0.3070	1.1847	0.7799	0.092*	
H19B	0.3884	1.0351	0.8315	0.092*	
H19C	0.2247	1.0805	0.9191	0.092*	
N21	0.6000 (3)	0.4588 (3)	0.7974 (3)	0.0328 (6)	
C21	0.5908 (5)	0.3401 (4)	0.7924 (4)	0.0477 (10)	
H21	0.5393	0.2745	0.8708	0.057*	
C22	0.6522 (5)	0.3064 (4)	0.6792 (4)	0.0490 (10)	
H22	0.6432	0.2190	0.6820	0.059*	
C23	0.7265 (4)	0.3989 (4)	0.5619 (3)	0.0322 (7)	
C24	0.7375 (6)	0.5215 (4)	0.5688 (4)	0.0505 (11)	
H24	0.7888	0.5889	0.4921	0.061*	
C25	0.6746 (5)	0.5472 (4)	0.6861 (4)	0.0479 (10)	
H25	0.6853	0.6324	0.6871	0.058*	
C26	0.7921 (4)	0.3663 (4)	0.4347 (3)	0.0400 (8)	
C27	0.6690 (9)	0.3075 (11)	0.4201 (8)	0.090 (3)	0.75
H27A	0.6344	0.2289	0.4980	0.134*	0.75
H27B	0.7117	0.2793	0.3424	0.134*	0.75
H27C	0.5827	0.3769	0.4105	0.134*	0.75
C28	0.8482 (16)	0.4831 (8)	0.3182 (6)	0.116 (5)	0.75
H28A	0.8887	0.4552	0.2404	0.174*	0.75
H28B	0.9289	0.5167	0.3281	0.174*	0.75
H28C	0.7643	0.5552	0.3086	0.174*	0.75
C29	0.9220 (9)	0.2496 (9)	0.4499 (7)	0.079 (2)	0.75
H29A	0.8831	0.1726	0.5280	0.119*	0.75
H29B	1.0048	0.2811	0.4588	0.119*	0.75
H29C	0.9601	0.2213	0.3724	0.119*	0.75
C27'	0.7280 (17)	0.4863 (16)	0.3290 (16)	0.040 (3)*	0.25
H27D	0.7455	0.5733	0.3275	0.060*	0.25
H27E	0.6186	0.4830	0.3518	0.060*	0.25
H27F	0.7803	0.4769	0.2425	0.060*	0.25
C28'	0.9670 (16)	0.3788 (17)	0.3825 (16)	0.043 (3)*	0.25
H28D	0.9843	0.4665	0.3793	0.065*	0.25
H28E	1.0094	0.3722	0.2944	0.065*	0.25
H28F	1.0168	0.3054	0.4411	0.065*	0.25
C29'	0.768 (2)	0.238 (2)	0.438 (2)	0.063 (5)*	0.25
H29D	0.8160	0.2303	0.3511	0.094*	0.25
H29E	0.6590	0.2316	0.4666	0.094*	0.25
H29F	0.8140	0.1651	0.5004	0.094*	0.25

### Atomic displacement parameters (Å^2^)

**Table:**

	*U*^11^	*U*^22^	*U*^33^	*U*^12^	*U*^13^	*U*^23^
Cd1	0.03329 (19)	0.02083 (18)	0.02336 (16)	−0.00619 (11)	−0.00577 (12)	−0.00565 (12)
N1	0.0426 (16)	0.0288 (16)	0.0430 (16)	−0.0125 (12)	−0.0074 (13)	−0.0095 (13)
C1	0.0312 (16)	0.0308 (19)	0.0447 (19)	−0.0041 (13)	−0.0115 (14)	−0.0107 (15)
S1	0.0540 (7)	0.0380 (7)	0.1310 (14)	−0.0182 (5)	−0.0373 (8)	0.0262 (7)
N11	0.0350 (14)	0.0263 (14)	0.0261 (12)	−0.0048 (11)	−0.0076 (10)	−0.0063 (11)
C11	0.0397 (18)	0.0288 (18)	0.0397 (18)	−0.0084 (13)	−0.0076 (14)	−0.0101 (14)
C12	0.0362 (18)	0.037 (2)	0.050 (2)	−0.0093 (14)	−0.0095 (15)	−0.0123 (17)
C13	0.0319 (17)	0.038 (2)	0.0422 (18)	−0.0007 (14)	−0.0098 (14)	−0.0136 (15)
C14	0.0352 (17)	0.0252 (17)	0.0291 (15)	−0.0020 (12)	−0.0071 (12)	−0.0072 (12)
C15	0.0339 (16)	0.0314 (18)	0.0279 (15)	−0.0043 (13)	−0.0069 (12)	−0.0086 (13)
C16	0.042 (2)	0.029 (2)	0.047 (2)	−0.0015 (14)	−0.0076 (16)	−0.0028 (15)
C17	0.099 (4)	0.049 (3)	0.044 (2)	−0.014 (3)	0.007 (3)	0.001 (2)
C18	0.062 (3)	0.033 (2)	0.082 (3)	0.0014 (19)	−0.032 (3)	0.001 (2)
C19	0.049 (2)	0.034 (2)	0.099 (4)	−0.0025 (17)	−0.020 (2)	−0.024 (2)
N21	0.0418 (16)	0.0299 (15)	0.0253 (12)	−0.0062 (11)	−0.0089 (11)	−0.0079 (11)
C21	0.069 (3)	0.033 (2)	0.0310 (17)	−0.0172 (17)	0.0013 (17)	−0.0092 (15)
C22	0.075 (3)	0.030 (2)	0.0347 (18)	−0.0162 (18)	0.0013 (18)	−0.0146 (15)
C23	0.0373 (17)	0.0295 (17)	0.0272 (14)	−0.0020 (13)	−0.0093 (13)	−0.0086 (13)
C24	0.080 (3)	0.039 (2)	0.0277 (17)	−0.028 (2)	−0.0004 (17)	−0.0092 (15)
C25	0.076 (3)	0.034 (2)	0.0332 (17)	−0.0239 (18)	−0.0063 (18)	−0.0113 (15)
C26	0.050 (2)	0.039 (2)	0.0310 (16)	−0.0035 (15)	−0.0087 (15)	−0.0165 (15)
C27	0.078 (5)	0.153 (9)	0.075 (5)	−0.032 (5)	−0.017 (4)	−0.073 (6)
C28	0.243 (14)	0.057 (5)	0.027 (3)	−0.056 (6)	0.006 (5)	−0.015 (3)
C29	0.073 (5)	0.098 (6)	0.062 (4)	0.023 (4)	−0.013 (4)	−0.048 (4)

### Geometric parameters (Å, º)

**Table:**

Cd1—N1^i^	2.301 (3)	C21—C22	1.385 (5)
Cd1—N1	2.301 (3)	C21—H21	0.9500
Cd1—N21	2.375 (3)	C22—C23	1.385 (5)
Cd1—N21^i^	2.375 (3)	C22—H22	0.9500
Cd1—N11^i^	2.403 (3)	C23—C24	1.382 (5)
Cd1—N11	2.403 (3)	C23—C26	1.527 (4)
N1—C1	1.155 (5)	C24—C25	1.385 (5)
C1—S1	1.618 (4)	C24—H24	0.9500
N11—C11	1.341 (5)	C25—H25	0.9500
N11—C15	1.342 (4)	C26—C29'	1.43 (2)
C11—C12	1.378 (6)	C26—C28	1.469 (8)
C11—H11	0.9500	C26—C27	1.542 (8)
C12—C13	1.388 (6)	C26—C29	1.548 (8)
C12—H12	0.9500	C26—C28'	1.564 (15)
C13—C14	1.389 (5)	C26—C27'	1.585 (16)
C13—H13	0.9500	C27—H27A	0.9800
C14—C15	1.402 (5)	C27—H27B	0.9800
C14—C16	1.532 (5)	C27—H27C	0.9800
C15—H15	0.9500	C28—H28A	0.9800
C16—C17	1.529 (6)	C28—H28B	0.9800
C16—C18	1.536 (6)	C28—H28C	0.9800
C16—C19	1.539 (7)	C29—H29A	0.9800
C17—H17A	0.9800	C29—H29B	0.9800
C17—H17B	0.9800	C29—H29C	0.9800
C17—H17C	0.9800	C27'—H27D	0.9800
C18—H18A	0.9800	C27'—H27E	0.9800
C18—H18B	0.9800	C27'—H27F	0.9800
C18—H18C	0.9800	C28'—H28D	0.9800
C19—H19A	0.9800	C28'—H28E	0.9800
C19—H19B	0.9800	C28'—H28F	0.9800
C19—H19C	0.9800	C29'—H29D	0.9800
N21—C21	1.325 (5)	C29'—H29E	0.9800
N21—C25	1.330 (5)	C29'—H29F	0.9800
			
N1^i^—Cd1—N1	180.000 (1)	N21—C21—H21	118.2
N1^i^—Cd1—N21	90.07 (11)	C22—C21—H21	118.2
N1—Cd1—N21	89.93 (11)	C23—C22—C21	120.5 (4)
N1^i^—Cd1—N21^i^	89.93 (11)	C23—C22—H22	119.7
N1—Cd1—N21^i^	90.07 (11)	C21—C22—H22	119.7
N21—Cd1—N21^i^	180.000 (1)	C24—C23—C22	115.3 (3)
N1^i^—Cd1—N11^i^	90.19 (11)	C24—C23—C26	122.8 (3)
N1—Cd1—N11^i^	89.81 (11)	C22—C23—C26	121.9 (3)
N21—Cd1—N11^i^	85.88 (10)	C23—C24—C25	120.8 (3)
N21^i^—Cd1—N11^i^	94.12 (10)	C23—C24—H24	119.6
N1^i^—Cd1—N11	89.81 (11)	C25—C24—H24	119.6
N1—Cd1—N11	90.19 (11)	N21—C25—C24	123.3 (3)
N21—Cd1—N11	94.12 (10)	N21—C25—H25	118.3
N21^i^—Cd1—N11	85.88 (10)	C24—C25—H25	118.3
N11^i^—Cd1—N11	180.00 (13)	C29'—C26—C23	117.8 (9)
C1—N1—Cd1	150.8 (3)	C28—C26—C23	113.7 (4)
N1—C1—S1	177.6 (4)	C28—C26—C27	110.8 (7)
C11—N11—C15	117.6 (3)	C23—C26—C27	108.3 (4)
C11—N11—Cd1	117.9 (2)	C28—C26—C29	110.3 (7)
C15—N11—Cd1	124.3 (2)	C23—C26—C29	107.9 (4)
N11—C11—C12	122.7 (3)	C27—C26—C29	105.5 (6)
N11—C11—H11	118.6	C29'—C26—C28'	108.9 (10)
C12—C11—H11	118.6	C23—C26—C28'	107.7 (6)
C11—C12—C13	119.0 (3)	C29'—C26—C27'	110.5 (11)
C11—C12—H12	120.5	C23—C26—C27'	107.0 (6)
C13—C12—H12	120.5	C28'—C26—C27'	104.0 (8)
C12—C13—C14	119.9 (3)	C26—C27—H27A	109.5
C12—C13—H13	120.0	C26—C27—H27B	109.5
C14—C13—H13	120.0	H27A—C27—H27B	109.5
C13—C14—C15	116.5 (3)	C26—C27—H27C	109.5
C13—C14—C16	123.4 (3)	H27A—C27—H27C	109.5
C15—C14—C16	120.0 (3)	H27B—C27—H27C	109.5
N11—C15—C14	124.1 (3)	C26—C28—H28A	109.5
N11—C15—H15	118.0	C26—C28—H28B	109.5
C14—C15—H15	118.0	H28A—C28—H28B	109.5
C17—C16—C14	109.1 (3)	C26—C28—H28C	109.5
C17—C16—C18	110.7 (4)	H28A—C28—H28C	109.5
C14—C16—C18	111.2 (3)	H28B—C28—H28C	109.5
C17—C16—C19	109.0 (4)	C26—C29—H29A	109.5
C14—C16—C19	109.3 (4)	C26—C29—H29B	109.5
C18—C16—C19	107.5 (4)	H29A—C29—H29B	109.5
C16—C17—H17A	109.5	C26—C29—H29C	109.5
C16—C17—H17B	109.5	H29A—C29—H29C	109.5
H17A—C17—H17B	109.5	H29B—C29—H29C	109.5
C16—C17—H17C	109.5	C26—C27'—H27D	109.5
H17A—C17—H17C	109.5	C26—C27'—H27E	109.5
H17B—C17—H17C	109.5	H27D—C27'—H27E	109.5
C16—C18—H18A	109.5	C26—C27'—H27F	109.5
C16—C18—H18B	109.5	H27D—C27'—H27F	109.5
H18A—C18—H18B	109.5	H27E—C27'—H27F	109.5
C16—C18—H18C	109.5	C26—C28'—H28D	109.5
H18A—C18—H18C	109.5	C26—C28'—H28E	109.5
H18B—C18—H18C	109.5	H28D—C28'—H28E	109.5
C16—C19—H19A	109.5	C26—C28'—H28F	109.5
C16—C19—H19B	109.5	H28D—C28'—H28F	109.5
H19A—C19—H19B	109.5	H28E—C28'—H28F	109.5
C16—C19—H19C	109.5	C26—C29'—H29D	109.5
H19A—C19—H19C	109.5	C26—C29'—H29E	109.5
H19B—C19—H19C	109.5	H29D—C29'—H29E	109.5
C21—N21—C25	116.4 (3)	C26—C29'—H29F	109.5
C21—N21—Cd1	120.2 (2)	H29D—C29'—H29F	109.5
C25—N21—Cd1	123.3 (2)	H29E—C29'—H29F	109.5
N21—C21—C22	123.6 (3)		

Symmetry code: (i) −*x*+1, −*y*+1, −*z*+2.
